# Non-diagnostic symptoms in a mouse model of autism in relation to neuroanatomy: *the BTBR strain reinvestigated*

**DOI:** 10.1038/s41398-018-0280-x

**Published:** 2018-10-26

**Authors:** Jamshid Faraji, Mitra Karimi, Cassandra Lawrence, Majid H. Mohajerani, Gerlinde A. S. Metz

**Affiliations:** 1 Canadian Centre for Behavioural Neuroscience, Department of Neuroscience, University of Lethbridge, Lethbridge, Canada; 2 Faculty of Nursing & Midwifery, Golestan University of Medical Sciences, Gorgan, Iran; 3 Iran Ministry of Education-Exceptional Education Organization, Inclusive-Integrated Education Program for Children with Special Needs, Tehran, Iran

## Abstract

Several mouse models of autism spectrum disorder (ASD), including the BTBR T + tf/J (BTBR) inbred strain, display a diverse array of behavioral deficits with particular face validity. Here we propose that phenotyping these preclinical models of ASD should avoid excessive reliance on appearance validity of the behavioral observations. BTBR mice were examined in three non-diagnostic symptoms modalities, beside an anatomical investigation for construct validity. The BTBR strain displayed poor sensorimotor integration as reflected by shorter stride length and greater latency on the balance beam task (BBT) when compared with C57BL/6 (B6) controls. Also, locomotor indices in the open-field task (OFT) revealed that BTBR mice traveled longer distances with a remarkably faster exploration than the B6 group in favor of hyperactivity and impulsiveness. Furthermore, analysis of spatial performance including search strategies in the Morris water task (MWT) indicated spatial impairment in the BTBR strain due to failure to employ spatial strategies during navigation. Quantitative cytoarchitectonics and volumetric examinations also indicated abnormal cortical and subcortical morphology in the BTBR mice. The results are discussed in relation to the neuroanatomical correlates of motor and cognitive impairments in the BTBR strain. We conclude that non-diagnostic autistic-like symptoms in the BTBR mouse strain can be impacted by autism risk factors in a similar way than the traditional diagnostic signs.

## Introduction

Autism spectrum disorder (ASD) is typically diagnosed in the early developmental period, and its main diagnostic criteria are behavioral, including a wide range of symptoms in personal, interpersonal, and communicational domains^1^. Patients with ASD show persistent deficits in social interaction, restricted interests and activities, and repetitive, stereotyped behaviors^2,3^. Given the highly diverse neuropathology and heterogeneous behavioral symptoms of ASD in humans, an animal model that can profile ASD-like characteristics with face validity appears useful for examining the potential relationship between neuropathology and behavioral abnormalities.

The use of rodents to model complex neuropsychiatric disorders with face validity proved to be challenging and comes with significant difficulties and limitations. Despite conceptual and procedural obstacles in developing animal models with core features of autism^4,5^, a subset of preclinical models using inbred mouse strains have been proposed in recent years that superficially express traits and behaviors often overlapping with the diagnostic criteria for clinical autism. Among the existing mouse models, the BTBR T + tf/J (Black and Tan Brachyury, BTBR) inbred mouse strain exhibits behavioral phenotypes analogous to core symptoms of autism, thus instituting reliable face validity for modeling ASD^6–8^. The BTBR mice exhibit social deficiencies characterized by poor social interaction and impaired communication, repetitive stereotype behaviors, and atypical vocalization^2,6,8–10^. Aside from behavioral symptoms, structural characteristics have also revealed neuroanatomical abnormalities such as complete or partial lack of inter-hemispheric connections in the corpus callosum and reduced hippocampal commissure associated with hippocampal malformation in the BTBR strain^7,11–15^.

In the light of the discussion concerning face validity, BTBR mice display wide-ranging social and emotional impairments analogous to three major domains of ASD diagnostic core symptoms^6,16^. Although productive and informative, face validity, or excessive reliance upon the appearance in preclinical trials to determine behavioral phenotypes, may confound original findings with biased, subjective interpretations. The latter poses a potential source of discrepancy between BTBR-relevant studies^5^. More importantly, heterogeneity of clinical presentation in individuals with ASD is a particular impediment^17,18^. This indicates that a wide variety of other non-diagnostic behavioral symptoms (e.g. impaired movement, poor coordination, hypo- or hyperactivity, low motivation, subtle deficits in spatial cognition together with learning difficulties) may not be considered as core symptoms in the animal models of ASD, including the BTBR strain. They are, however, still prominent in a substantial fraction of the clinical population with ASD. Accordingly, beyond face validity alternative explanations for the presence of specific behaviors should be investigated, particularly for those which are less probed or can be linked to brain morphology. In the present study, balance and coordination, locomotor activity, and spatial performance are explored in relation to neuroanatomical correlates in BTBR mice to explore further preclinical aspects of this ASD animal model.

## Materials and methods

### Animals

Twenty-two male and female mice (C57BL/6 [B6]; *n* = 13 and BTBR T + tf/J [BTBR]; *n* = 9), 3–4 months old were used in this study. No statistical methods were used to predetermine sample size, and the ample sizes are comparable to common standards reported in the field^19,20^. BTBR mice were obtained from Jackson Laboratory (Bar Harbor, ME, USA). Animals were housed at room temperature (21–24 °C) on a 12-h light/dark cycle (lights on at 7:30) with ad libitum access to food and water, and were handled for approximately 3–4 min daily for 5 consecutive days prior to behavioral testing. All procedures in this study were approved by the University of Lethbridge Animal Care Committee in compliance with the standards set forth by the Canadian Council for Animal Care. No randomization was applied to employ the BTBR mice. The number of animals per group used in the present study was the maximum that was approved by the University of Lethbridge Animal Care Committee, and was high enough to ensure statistical power of the analyses that were used. According to ethical regulations, one BTBR mouse was excluded from behavioral testing due to severe self-injurious behaviors.

### Behavioral assessment

In the present experiment, the order of testing for behavioral assessment was: (1) balance beam task (BBT); (2) open-field task (OFT); and (3) Morris water task (MWT). The task order was randomized and all animals were allowed to rest for nearly 24 h after being tested in each behavioral task.

#### Balance beam task

The BBT was employed to test sensorimotor integration (i.e. coordination and balance^21^; with modifications). Animals were positioned on one end of an aluminum round bar (1 cm wide, 92 cm long, and 50 cm high) and their home cage was located at the other end of the bar. A foam pad was placed underneath to cushion falling animals. The animals were tested for at least three trials on the bar, and their movements were video recorded from a lateral view using a digital camcorder (Panasonic HDC-SDT750) at 60 frames/s with an exposure rate of 1 ms. The latency to traverse the bar, the number of times the hind feet slipped off the bar and stride length were recorded. Each stride was defined as the distance between the takeoff and landing positions of the left hindlimb. A high-contrast point with proper vertical and horizontal edge definition was chosen on the back of the hindlimb (Sony Vegas Pro 11, Japan). Stride length (cm) on the beam was measured by the number of pixels in the tracked frames traced between the takeoff and landing positions. Also, a suitable target region for tracking was determined based on a pattern that was clearly visible in every frame. If the target point did not contain a high-contrast point to track, the preprocess parameters (e.g. increasing the contrast) were adjusted to make the source image easier to track.

#### Open-field task

The OFT was used to assess psychomotor outcomes and exploratory behavior^22^. The task (a 154 cm diameter white circular tub, elevated 40 cm above the floor) was used under dim illumination. Each animal was individually placed in the central zone of the OFT and video recorded for 20 min with a camera mounted above the arena. The experimenter left the room immediately after placing the mouse in the task. Video recordings were analyzed for overall path length and path speed, path length taken by animals in the central zone (~52 cm diameter), and the number of stops by the computer tracking system (HVS Image 2020, UK). Specifically, path length and path speed were measured as an indicator of motivation level. After each animal, the apparatus was cleaned with 70% alcohol. A 5–7 min interval between cleaning and the start of the next testing session was set for alcohol odor dissipation.

#### Morris water task

The MWT was used to assess place and spatial learning^23^. The task consisted of a circular pool (154 cm diameter) filled to within 20 cm of the top of the wall with water (22 ± 1 °C). The pool was located in a room enriched with distinct distal cues, which remained unobstructed throughout the duration of the experiment. A circular escape platform (12 cm radius) was visible above the water level or submerged below the water surface and located at any one of four quadrants. The platform was positioned half-way between the center and the pool wall. The animals were introduced into the water facing the pool wall at defined starting positions. All animals were required to locate the platform using either distal and/or proximal cues during navigation in the task. Each testing trial began with the mouse being placed in the pool at one of the four cardinal compass positions (North, West, South, and East) around the perimeter of the pool according to a pseudo-random sequence. The maximum duration of each swim trial was 60 s, and if a mouse found the platform within the allotted time, it was allowed to remain on the platform for 5 additional seconds. If it did not find the platform during the selected time, then it was placed onto the platform for 10 s by the experimenter before being placed back into its holding cage.

#### MWT testing protocol

##### Day 1: visible platform

Because performance in the MWT may be affected by deficits in non-cognitive domains, animals were tested by a 1-day testing protocol (six trials per animal) using a visible (cued) platform. The platform was elevated above the water surface (0.5–1 cm) and was marked with black electrical tape. The location of the visible platform (quadrant 1) remained constant from trial to trial.

##### Day 2: hidden platform

Animals were required to find the hidden (invisible) platform across four 60-s trials in the task. The platform was submerged 0.5–1 cm below the water level, and was camouflaged by adding non-toxic white paint (Craft Smart, TX, USA) to the water. The platform also moved to a new location (quadrant 3), and testing was not finished until four different locations were completed by each animal. The protocol allowed animals to encode the goal spatial location within a multiple-trials framework

##### Days 3–7: hidden platform

The testing procedures used in this phase were identical to those described in the previous phase with the exception that mice were required to locate the hidden platform in a new quadrant (quadrant 4) for 5 consecutive days. Also, the location of the hidden platform remained constant across the testing days. The platform-reversal protocol assumes that animals establish new spatial relationships between spatial contexts and the new platform position after they have previously learned to navigate to a given goal position. Search skills obtained in the former spatial trials may also contribute in developing new search strategies with more cognitive flexibility to explore the platform in the new position. Thus, during the platform-reversal testing, the previous spatial configuration will be updated to establish more efficient navigation within the new spatial context and relationships

Movements of the animals during the place and spatial learning including latency (time spent to find the platform), swim length, swim speed, error index (swim error or corridor percent path), and path efficiency ratio (actual path length divided by direct path length [Euclidean distance between the starting point and platform]) were recorded and analyzed by an image-computerized tracking system (HVS Image 2020, UK). The error index in the present study shown in path percentage in the corridor refers to the accuracy of a mouse swim trajectory within a 20-cm-wide corridor from the start point to the platform. Any deviation from this corridor during swimming was scored as an error^24^.

##### Day 8: probe trial

Experimental groups were also subjected to probe trial testing on the eighth day of the MWT testing. This served as a transfer test to determine the extent to which the mice had learned about the location of the platform. The platform was removed from the pool and the animals were allowed to swim freely for 30 s. The percentage of time that they spent in each quadrant of the task including the target quadrant (quadrant 4) was recorded^25^. Here spatial reference memory was determined by significantly greater search time in the target quadrant compared to other quadrants of the task.

Also, because the conventional parameters of the MWT alone are not sufficient to draw a reliable conclusion on the hippocampal-dependent aspects of spatial performance^26^, the overall search strategies or patterns of each mouse during navigation in the task were analyzed. Two separate investigators blind to the animals genotypes and grouping did manually categorize (modified from refs. ^27–32^) the predominant search strategies used by the mice during each trial. A certain strategy was chosen based upon swim path plotted by the tracking system and calculation of pool coverage as well as time spent in a specific area of the pool. The inter-rater reliability (level of agreement between raters) on strategy categorization was 91%. Search strategies in the present experiment were classified as follows: (1) non-spatial strategies including thigmotaxis (T; wall-hugging behavior or a repetitive circular pattern of swimming near to the wall), random swim (RS; swimming pattern with no spatial preference), scanning (S; searching the central or inner zone), and chaining (C; circular swimming in the inner and outer zones without looping); and (2) spatial strategies including directed search (DSe; remaining in the corridor toward the platform), focal search (FS; swimming directly to and searching for the platform in the target quadrant), direct swim (DSw; swimming directly to the platform), and perseverance (P; scanning or searching for the platform in a non-target quadrant).

### Gross anatomy and histological assessment

Animals in both groups were euthanized and intracardially perfused when behavioral testing completed. Brains including cerebellum were removed, weighed, and fixed for coronal sectioning and cresyl violet staining. Criteria for brain abnormality was chosen based on the brain weight after extraction^33^, and no animal was excluded from analysis for abnormal brain weight. The stained sections were imaged using a Nanozoomer 2.0RS slide scanner (Hamamatsu, Japan) for histological analysis and presentation. From the coronal sections, measurements included cortical and dorsal hippocampal (dHPC) volumetrics, neuronal density, and cortical thickness in both hemispheres. Furthermore, in order to enhance the accuracy of the histology, six animals from B6 group and one animal from the BTBR group were excluded from histological assessments and also from all other corresponding volumetric analyses because of damages to one or both hemispheres, and technical issues such as missing and/or damaged sections.

#### Volume analysis

For each animal (B6, *n* = 7; BTBR, *n* = 8), a set of eight cross sections stained with cresyl violet was considered for cortical volumetric analysis. The most rostral section measured for cortical volume was located at ~1.78 mm anterior to bregma and the most caudal section at −2.06 mm posterior to bregma. The volume averages were calculated by dividing the sum of measures obtained from each brain by the total number of sections (shrunk brain area in mm^2^). The approximate volume of the cortex and hippocampus (HPC; shrunk volume in mm^3^) was determined by multiplying the total area in mm^2^ by both the thickness of each slice (40 μm) and the sampling interval (5)^34^. The hippocampal volume (B6, *n* = 7; BTBR, *n* = 8) in each mouse was estimated using a set of four cross sections of the dHPC area, from ~−1.06 to ~−2.06 mm relative to bregma. In the case of missing or damaged sections (less than three sections for each mouse) data were calculated as average area values from preceding and following sections. For each tissue section, the contours of the bilateral hemispheres were traced and their areas were measured using ImageJ 1.47b (http://imagej.nih.gov/ij; NIH, USA).

#### Cortical thickness

Three points (dorsal, lateral, and ventral) from each brain and hemisphere were selected for cortical thickness^35^. The most rostral section measured was located at ∼2.10 mm anterior to bregma and the most caudal section at ∼−0.46 mm posterior to bregma. For each point, a vector was considered from the tangent of the outer edge to the inner edge of the cortex. The NDP.view2 viewing software U-12388-01 (Hamamatsu, Japan) was used to record up to six measurements of cortical thickness from each coronal section, three from each hemisphere. Cortical thickness in the present experiment was a mean measure of both hemispheres in seven consecutive slices.

#### Cellular density and cytoarchitectonics

Cellular density analysis (quantitative cytoarchitectonics) was performed using ImageJ 1.47b (http://imagej.nih.gov/ij; NIH, USA). Two regions of interest (ROI; dorsal [~0.352 mm^2^] and lateral [~0.30 mm^2^]) were determined in each hemisphere. Both left and right ROIs included the same cortical regions and mainly all six cortical layers. Four approximate planes (between anterior-posterior [AP] ∼1.98 and ∼0.74 mm) of stained sections in each brain were selected. For cell counting, the red, green and blue (RGB) images were converted to 16-bit greyscale images by ImageJ, and a threshold of 90 and 180 was set for black/white background, respectively. When all particles highlighted, the particles that have merged together, were separated by Binary (watershed) option. This can often accurately cut the attached particles apart by adding a 1-pixel thick line where it seems the division should be. The binary image of the particles in black/white ROIs were counted for both hemispheres by the Analyze Particle program. It should be pointed out that because the size and circularity of the particles may affect the final counts, the size and circularity of particles in the present study were set on pixels (pixle^2) 0-infinity and 0.00–1.00, respectively. An analysis of particles within the ROIs (dorsal and lateral) was separately performed for each section, hemisphere and region. All histological assessments were performed by the same person, who was blind to the groups’ assignment.

### Data analysis

Because the data in the present study were not normally distributed, Kruskal-Wallis *H* test, a rank-based nonparametric test for analysis of variance was used to detect differences between means of groups across multiple test attempts (e.g. trials, days, regions, and quadrants). The Mann-Whitney *U* test was also used to compare means of the two groups for a single dependent variable with Bonferroni correction for multiple comparisons when necessary. Correlation between variables (cell density, volumes, latency, spatial strategy, etc.) was analyzed by Spearman’s rank correlation. In all statistical analyses (SPSS 16.0, SPSS Inc., USA), a *p*-value of <0.05 (two-tailed) was chosen as the significance level, and results are presented as mean ± standard error.

## Results

### Behavioral assessment

#### Sensorimotor integration in the BBT

**Fig. 1:**
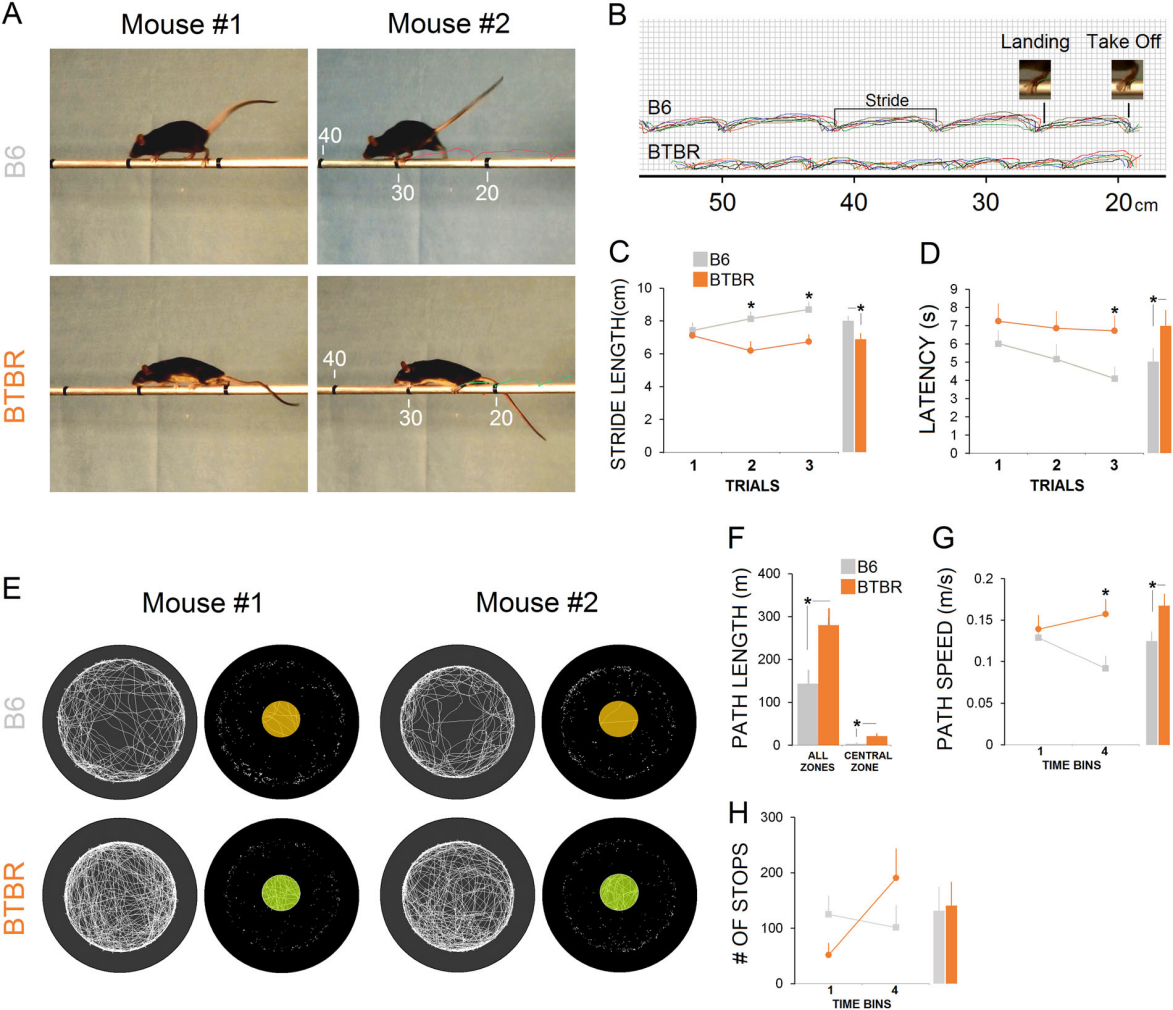
Assessment of motor coordination on the balance beam task (BBT) and exploratory behavior within the open-field task (OFT). **a** Representative illustration of beam walking for two animals from B6 and BTBR groups. Although all animals were able to stay and move on the balance beam, the BTBR group was unable to perform successful steps on the bar. Stiff body, crawling position, flat back, and fallen tails that may represent compensatory mechanisms to support the stepping pattern. **b** Illustration of motion tracks constructed from six animals in each group (each colored motion track represents one animal). Inset pictures display takeoff and landing positions in each stride. Note that stride length, the pixel-based distance between takeoff and landing positions of the left hindlimb, was shorter in BTBR than in B6 animals. **c** B6 mice showed significantly longer stride length on the BBT compared with BTBR animals in trials 2 and 3 (*n* = 9/group). Average stride length (cm) is shown in bar graphs. **d** Latency, the traverse time, indicated a difference between B6 and BTBR groups in trial 3. Average latency in in the B6 and BTBR animals is shown in bar graphs. No differences were found in foot slips (data not shown). **e** Path taken by two representative mice from B6 and BTBR groups in the OFT during a 20-min test session. Note the differences between the paths occurred in the central zone (yellow and green circles; ~52 cm diameter) in both groups. White dots indicate the number of stops during exploration in the arena. **f** The average path length taken by BTBR mice was longer than in the B6 group, even in the central zone of the open field. **g** Although not different in the first time bin, on average, BTBR mice explored the arena faster than B6 mice (bar graphs). **h** The average number of stops made by mice in both groups during the 20-min exploration was not different. Asterisks indicate significant difference between groups: *p ≤ 0.05. Error bars show ± SEM

All animals were able to stay and move on the balance beam (Fig. 1a). However, B6 mice (*n* = 9) made significantly greater average stride length compared with BTBR animals (*n* = 9; B6: 8.07 ± 0.43 cm vs. BTBR: 6.66 ± 0.51 cm; *U* = 21.500, *p* ≤ 0.039, Mann-Whitney *U*; Fig. 1b, c), particularly on trials 2 and 3 (all *p* ≤ 0.05). More importantly, the average stride length in B6 mice increased across three trials (7.41 < 8.12 < 8.69 cm) suggesting that they gradually were able to reach better balance and perform longer steps across repeated trials. This profile of movement, however, was the reverse in the BTBR mice in the last trial (Fig. 1c). Also, latency (i.e. time spent to traverse the bar) showed that B6 mice were able to cross the bar with shorter latency when compared to the BTBR group (B6: 5.09 ± 0.74 s vs. BTBR: 6.94 ± 0.91 s; *U* = 18, *p* ≤ 0.041, Mann-Whitney *U*; Fig. 1d; Supplementary Movies 1, 2). A significant correlation was found between stride length and latency on the BBT (B6: *r*_s_ = −0.614, *p* ≤ 0.039; BTBR: *r*_s_ = −0.466, *p* ≤ 0.041). Accordingly, a larger stride length in the B6 group was associated with lower latency during beam walking. In contrast, because BTBR animals displayed shorter stride length, they had higher latency than B6 animals when crossing the beam. Moreover, despite a slight increase of hindlimb average slips across all trials in the B6 group, no significant difference was found between groups in the number of foot slips (4 vs. 2, *p* ≥ 0.077, Mann-Whitney *U*; data not shown).

#### Exploratory behavior in the OFT

Three locomotor indices included overall path length (m) and path speed (m/s) for motivational status, and path taken by animals in the central zone of the open field along with the number of stops to assess emotionality. Representative activity traces and parameters in the open field are shown in Fig. 1e for two mice of each B6 and BTBR groups (*n* = 13 and 9, respectively). Path length (distance traveled) shown by both groups indicated that the BTBR group traveled significantly more distance than B6 mice (B6: 143.69 ± 31 m vs. BTBR: 280.5 ± 39 m; *U* = 23.500, *p* ≤ 0.028, Mann-Whitney *U*) even within the central zone of the open field (B6: 3.45 [2.40%] ± 0.94 m vs. BTBR: 21 [7.48%] ± 0.4.96 m; *U* = 16, *p* ≤ 0.043, Mann-Whitney *U*; Fig. 1f). Compared to B6 mice, the BTBR group also explored the open field faster. However, between-group difference was significant only for the fourth time bin (i.e. the last five minutes; B6: 0.091 ± 0.01 m/s vs. BTBR: 0.157 ± 0.018 m/s; *U* = 21, *p* ≤ 0.033, Mann-Whitney *U*; Fig. 1g; Supplementary Movies 3, 4). No significant difference in the number of stops was found between groups in any of the four time bins (all *p* ≥ 0.05, Mann-Whitney *U*; Fig. 1h) despite slightly reduced stops in the BTBR group.

#### Spatial performance in the MWT: latency and speed

**Fig. 2:**
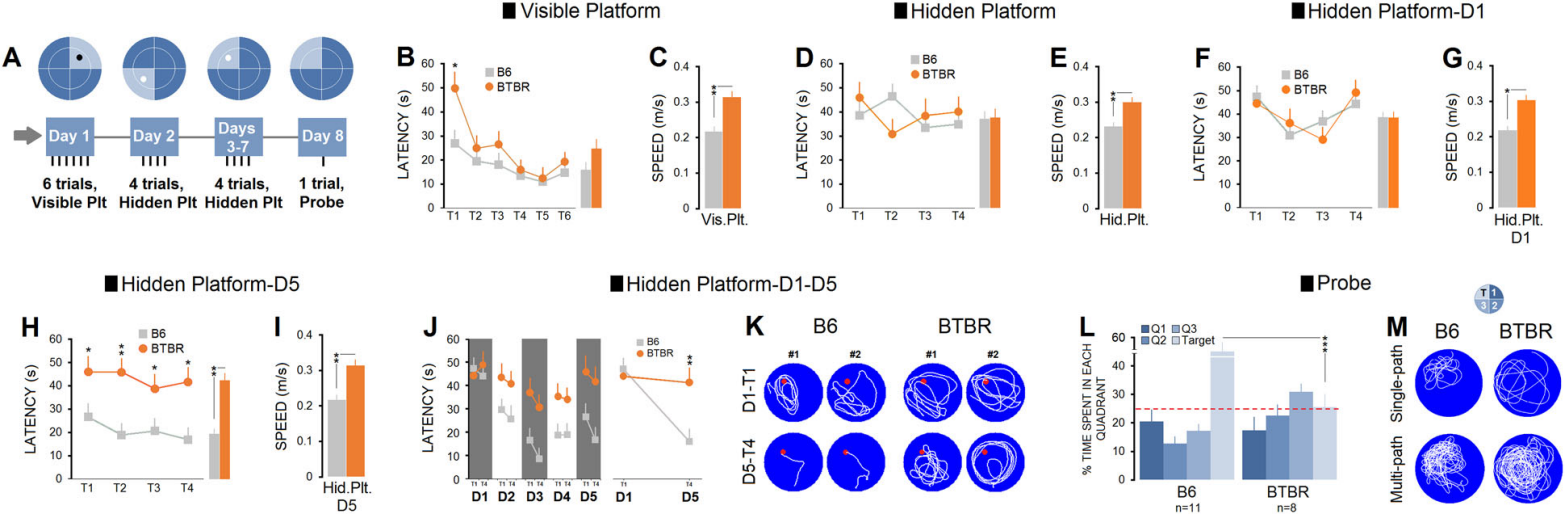
Examination of spatial performance using conventional learning parameters in the Morris water task (MWT). **a** Experimental design to assess working and reference memory in mice. **b**, **c** Latency in the MWT using a 1-day testing protocol for non-spatial learning showed that all animals (B6: *n* = 11, BTBR: *n* = 8) were able to learn the location of the visible platform, although BTBR mice significantly swam faster than B6 group during navigation. **d**, **e** Mean latency in the 1-day testing protocol for spatial performance revealed no group differences. Similar to the results obtained in the visible trial (**b**, **c**), mice in the BTBR group significantly swam faster than B6 mice. **f**, **g** Latency on the first day of the platform-reversal protocol for spatial learning and memory did show no significant difference between groups across four trials indicating that both groups showed the same patterns of spatial navigation within the task. The BTBR group was still faster than B6 group in locating the hidden platform. **h**, **i** However, spatial performance profile on the last day of the platform-reversal protocol (day 5) was significantly different: B6 animals, although faster in swimming displayed impaired spatial working memory across all testing trials when compared to BTBRs. **j** Latency across 5 days of the platform-reversal protocol indicated that only B6 mice were able to employ the search skills obtained in the previous spatial trials to explore the hidden platform in the new position. **k** Representative swim paths of two B6 and BTBR mice across testing trials (trials 1 and 4) within the MWT. Red spots indicate the location of the hidden platform. BTBR mice obviously displayed different search strategies even during the last trials by showing more “chaining” and “scanning”, both non-spatial strategies than B6 mice. **l** The mean percentage of time spent in four quadrants of the task during the probe trial after completion of the hidden platform-reversal paradigm. Mice in BTBR group showed no preference to spend time in the target quadrant (Q4) indicating impaired reference memory. **m** Representative probe trial trajectories illustrating focal search within the target quadrant only in B6 mice. Single-path plots represent one mouse from each group, and multi-path plots depict path taken by all animals in each group (B6: *n* = 11, BTBR: *n* = 8). Asterisks indicate significant differences: *p ≤ 0.05; **p ≤ 0.01. Error bars show ± SEM

Spatial performance was tested by an 8-day assessment protocol (Fig. 2a). Because latency and path length always reveal a similar profile of spatial learning^25,36,37^, and latency can also be potentially affected by differences in swim speed^26^, in the present study only latency and speed were considered for preliminary spatial analysis.

##### Visible-platform assessment (1 day)

The latency in both groups decreased over six trials of non-spatial testing suggesting that all mice, regardless of their experimental conditions were able to locate the cued platform at a similar rate (B6: 17.3 ± 2.63 s vs. BTBR: 24.84 ± 3.17 s; *n* = 13 and 9). No significant difference was found between groups in trials 2–6 (all *p* ≥ 0.05), except the first trial (*χ*^2^ (1) = 5.900, *p* ≤ 0.015, Kruskal-Wallis with a mean rank of 8.77 for B6 and 15.44 for BTBR groups; Fig. 2b). However, BTBR mice swam consistently faster than B6 mice (0.314 ± 0.016 m/s vs. 0.217 ± 0.014 m/s, *U* = 19.500, *p* ≤ 0.009, Mann-Whitney *U*; Fig. 2c) in most trials including trial 1 (all *p* ≤ 0.05, Kruskal-Wallis with a mean rank speed of 7.38 for B6 and 17.44 for BTBR mice).

##### Hidden-platform assessment (1 day)

A single testing day for spatial function was performed over four trials with the hidden platform located in quadrant 3. Both B6 and BTBR groups (*n* = 13 and 9) acquired and retrieved the location of the hidden platform in a similar manner (B6: 38.36 ± 2.67 s vs. BTBR: 38.81 ± 3.21 s; all *p* ≥ 0.05, Kruskal-Wallis with a mean rank latency of 11.39 for B6 and 11.65 for BTBR groups; Fig. 2d). However, BTBR mice swam significantly faster than the B6 group during spatial navigation (B6: 0.231 ± 0.011 m/s vs. BTBR: 0.3 ± 0.014 m/s, *U* = 18, *p* ≤ 0.007, Mann-Whitney *U*; Fig. 2e).

##### Hidden-platform assessment (5 days)

Day 1: In all trials, both groups (*n* = 13 and 9) displayed relatively the same profile of spatial ability (Fig. 2f), and no significant difference was found between groups in terms of the time spent to find the hidden platform (all trials *p* ≥ 0.05, Kruskal-Wallis). The BTBR mice, however, moved consistently and significantly faster than B6 mice (B6: 0.218 ± 0.012 m/s vs. BTBR: 0.303 ± 0.015 m/s, *U* = 19.500, *p* ≤ 0.007, Mann-Whitney *U*; Fig. 2g).

Day 5: Spatial assessment on day 5 indicated that BTBR mice located the platform more slowly than B6 animals (all trials *p* ≤ 0.05, Kruskal-Wallis; Fig. 2h). Moreover, BTBR mice moved consistently faster than B6 mice during spatial navigation in all trials (B6: 0.217 ± 0.014 m/s vs. BTBR: 0.314 ± 0.017 m/s, *U* = 4, *p* ≤ 0.001, Mann-Whitney *U*; Fig. 2i).

In summary, the 5-day hidden platform assessment using latency and swim speed showed that only the B6 group acquired and retrieved the spatial location. In contrast, BTBR mice, although faster in swimming, displayed a substantial impairment in spatial performance. Although increased swimming speed in the MWT may provide a major contribution to a reduced latency, results in the present experiment indicate that the possibility cannot be precluded that animals in the faster group (BTBR) will necessarily perform more accurately because their latency to find the hidden platform particularly on days 2–5 reflect a poor spatial function compared to B6 mice.

Changes in latency over 5 days of acquisition (days 1–5; fixed platform location) are depicted in Fig. 2j. Further analysis of latency to find the hidden platform in the first trial of day 1 indicated that both groups were able to acquire and retrieve the spatial information at a similar rate (*p* = 0.412, Kruskal-Wallis). However, although all rats showed a gradual decrease in the latency in the last trial of day 5, B6 mice located the platform more quickly than BTBR mice (B6: 16.87 ± 5.38 s vs. BTBR: 41.74 ± 6.47 s; all *p* ≤ 0.023, Kruskal-Wallis with a mean rank latency of 8.88 for B6 and 15.28 for BTBR groups). Representative swim paths of two B6 and BTBR mice across the two trials (day 1-trial 1 and day 5-trial 4) are shown in Fig. 2k).

#### Probe function

B6 group (*n* = 11) spent a considerable proportion of their time (49.30%) searching in the training (target) quadrant (quadrant 4) in which the hidden platform had previously been located. In contrast, the BTBR mice (*n* = 8) exhibited a more diffuse pattern of searching, with much less spatial bias toward the former training quadrant (25.92%; *p* ≤ 0.004, Kruskal-Wallis with a mean rank percent time of 13.18 for B6 and 5.62 for BTBR groups; Fig. 2l). The representative plots of single path of one mouse from each group along with plots of multi-path for each group are presented in Fig. 2m). In summary, only B6 mice preferentially swam in quadrant 4 in which the hidden platform had previously been offered to both groups during the testing days.

#### Corridor percent path

**Fig. 3:**
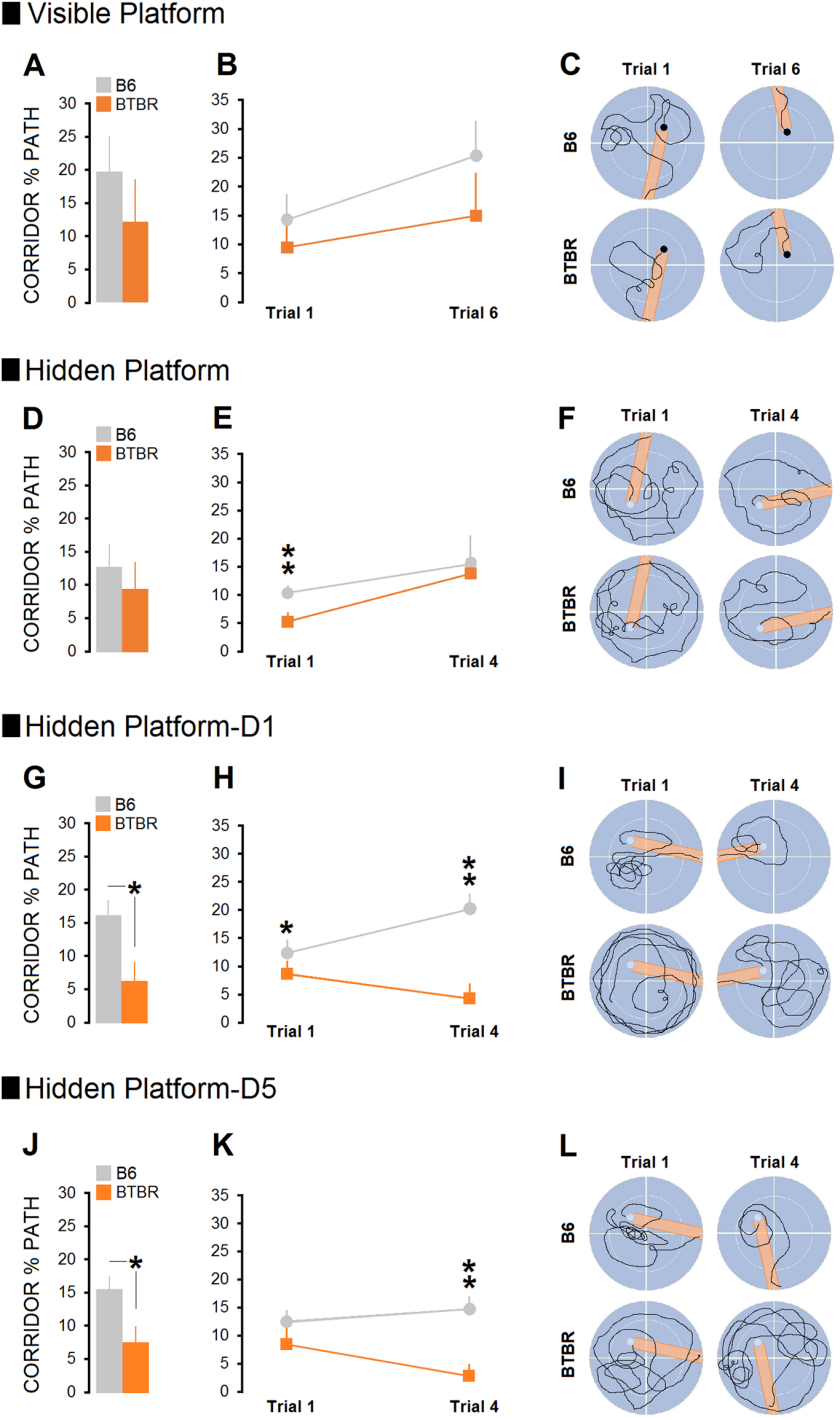
Examination of spatial performance using corridor percent path (Whishaw’s error index) in the MWT. Left row: **a**, **d**, **g**, **j** average corridor percent path indicate inaccurate swims relative to the platform location only in the platform-reversal protocol in BTBR mice. Analysis of spatial error via corridor percent path showed that both groups displayed the same pattern of swim to the platform on the visible-platform assessment day. Middle row: **b**, **e**, **h**, **k** however, trial-by-trial analysis of the corridor percent path indicated that in-corridor swimming of B6 mice was significantly more than BTBR group in the first trial of the single-day protocol for spatial testing. When tested in the platform-reversal protocol for 5 days when animals were required to locate the hidden platform in the new position, BTBR group swam significantly less than B6 mice in the corridor to the platform. Right row: **c**, **f**, **i**, **l** representative swim paths show corridor errors in the first and last trials made by mice during non-spatial and spatial navigations. Orange strips in plots represent required swim corridors (20 cm) to the platform (B6: *n* = 13, BTBR: *n* = 9). Asterisks indicate significant differences: *p ≤ 0.05; **p ≤ 0.01. Error bars show ± SEM

Figure 3a–l shows corridor percent time or swim error made by B6 (*n* = 13) and BTBR (*n* = 9) groups over 7 testing days.

##### Visible-platform assessment (1 day)

Analysis of spatial error via corridor percent time in the MWT indicated that both groups displayed a similar swim pattern. No significant difference was found between groups (all *p* ≥ 0.05, Kruskal-Wallis; Fig. 3a, c).

##### Hidden-platform assessment (1 day)

There was no overall difference between groups on the single-day testing with hidden platform for spatial error, although B6 mice remained significantly more in the corridor during swimming to the platform in trial one (B6: 10.35 ± 1.29% vs. BTBR: 5.3 ± 1.55%; *p* ≤ 0.006, Kruskal-Wallis with a mean rank corridor percent path of 14.69 for B6 and 6.89 for BTBR groups; Fig. 3d–f).

##### Hidden-platform assessment (5 days)

Day 1: An overall difference was observed between groups on the first day of the hidden-platform assessment (B6: 16.30 ± 2.19% vs. BTBR: 6.44 ± 2.14%; *U* = 6.5, *p* ≤ 0.03, Mann-Whitney *U*). Trial-by-trial analysis of swimming error also showed that B6 mice remained more in the corridor than BTBR mice during swimming to the hidden platform in the first trial (B6: 12.34 ± 2.47% vs. BTBR: 8.6 ± 2.34%; *p* ≤ 0.005, Kruskal-Wallis with a mean rank corridor percent path of 14.77 for B6 and 6.78 for BTBR groups) and last trials (B6: 20.26 ± 2.63% vs. BTBR: 4.28 ± 2.62%; *p* ≤ 0.032, Kruskal-Wallis with a mean rank corridor percent path of 13.96 for B6 and 7.94 for BTBR groups; Fig. 3g–i).

Day 5: Again, an overall significant difference was found between groups (B6: 15.70 ± 2.27% vs. BTBR: 7.72 ± 2.43%; *U* = 9.50, *p* ≤ 0.04, Mann-Whitney *U*) where B6 animals swam significantly more in the corridor than BTBR group in trials 3 and 4 on the last day of spatial testing (all *p* ≤ 0.05, Kruskal-Wallis; Fig. 3j–l).

#### Path efficiency ratio

**Fig. 4:**
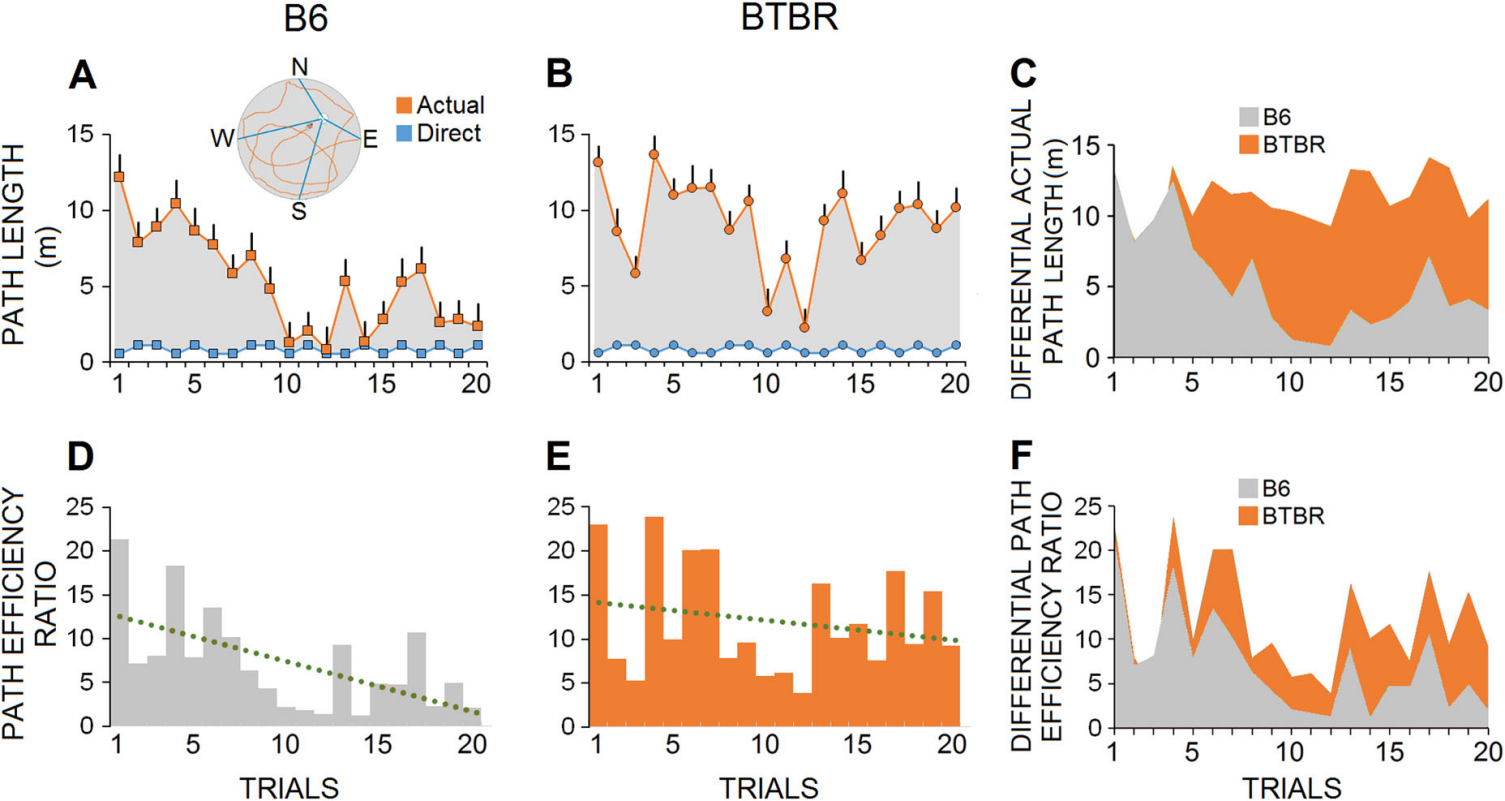
Path efficiency ratio in the MWT. **a**, **b** The length of actual paths taken by two individual mice from each group show that BTBR mouse was not able to reduce path length to the level of B6 mouse. A bi-phasic (hump-shape) curve of path length was prominent in BTBR mice across the 20 trials of spatial navigation. **c** Comparative average actual path indicated an obvious difference between B6 and BTBR mice starting from trial five (B6: *n* = 13, BTBR: *n* = 9). **d**, **e** Furthermore, path efficiency ratio (the ratio of the actual path to the optimal path) as a measure for efficiency of the search strategies also showed higher ratio in the BTBR mice indicating the impaired spatial accuracy in this group. **f** The average differential path efficiency ratio compares search accuracy in both groups throughout training

The efficiency of the search strategies employed for reaching the hidden platform was analyzed by calculating the path efficiency ratio, the ratio of the direct (optimal) path to the actual path taken by animals. The BTBR mice were unable to reduce their path length to the level of B6 mice, except in the first four trials of spatial training (BTBR: 11.15 ± 0.45 m vs. B6: 5.24 ± 0.80 m, Fig. 4a–c). Specifically, the BTBR group exhibited a bi-phasic (hump-shape) curve of path length, which was formed from trial 3 to trial 20. In contrast, B6 mice started spatial navigation with a left-skewed uni-phase length curve by trial 4, which continued to the last trials (*U* = 38, *p* ≤ 0.000, Mann-Whitney *U*; Fig. 4c). Moreover, compared with B6 mice, the BTBR animals displayed a larger path efficiency ratio in most trials (Fig. 4d–f) indicating that spatial accuracy was severely impaired in this group (B6: 7.14 ± 1.24 vs. BTBR: 12.05 ± 1.34, *U* = 104, *p* ≤ 0.009, Mann-Whitney *U*; Fig. 4f).

#### Search strategies and improvement rate

##### Search strategy

**Fig. 5:**
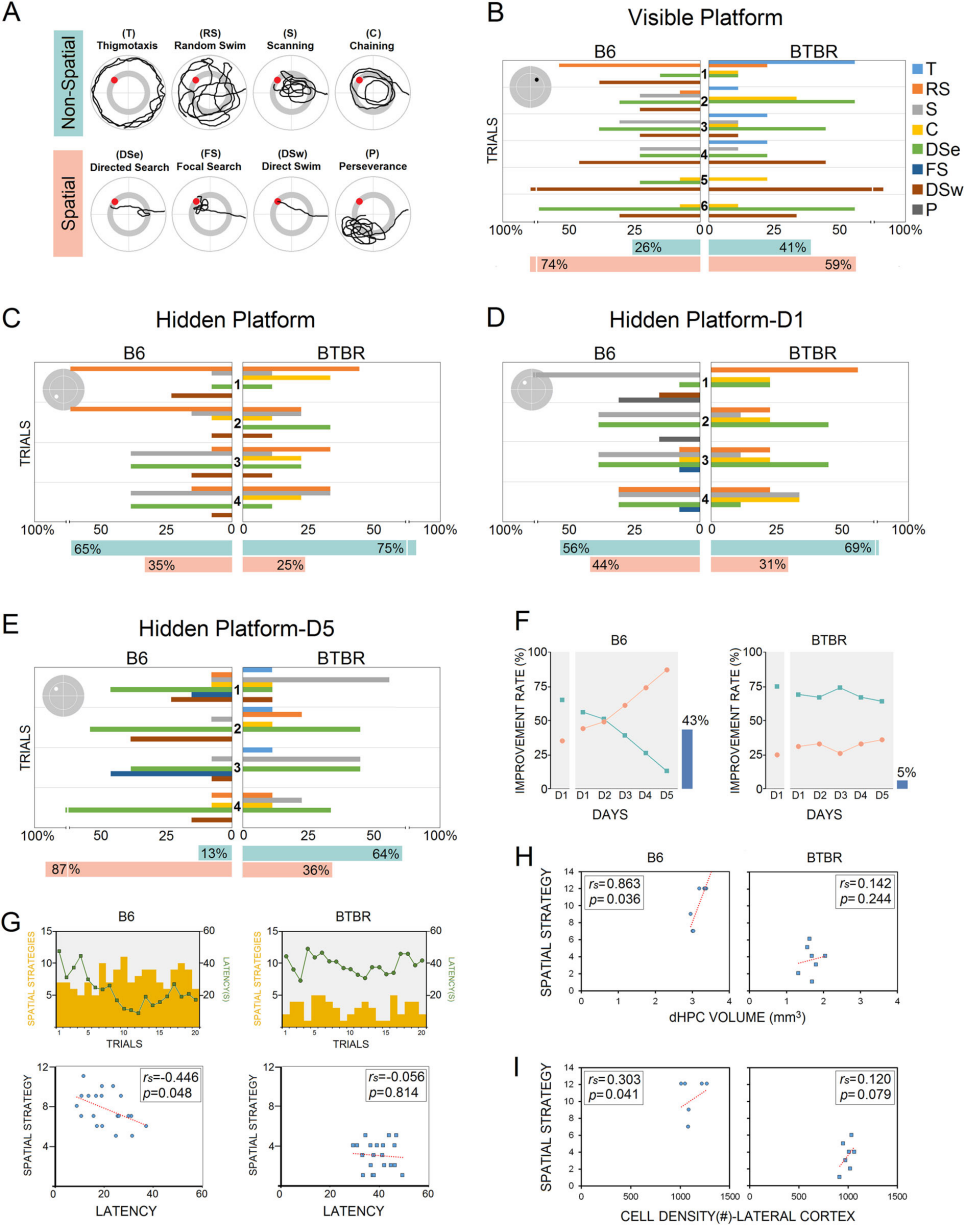
Search strategies used by B6 and BTBR mice in the MWT. **a** A schematic representation of eight distinct types of swimming strategies employed by mice during navigation in the task. Swim strategies are categorized as non-spatial and spatial search strategies. **b**–**e** Analysis of strategies showed that only B6 group displayed a progression toward an increase in the use of spatial strategies with training. Note the changes in the percentage of spatial strategies employed by B6 mice in the hidden platform-reversal paradigm (**d**, **e**). **f** Percentage difference in the improvement rate in the B6 (43%) relative to BTBR mice (5%) during spatial navigation revealed that BTBR mice were not able to establish new spatial relationship between spatial contexts and the new platform position in the hidden platform-reversal paradigm after they have previously learned to navigate to a given goal position. **g** A significant reduction in latency was accompanied by a significant increase in the use of spatial search strategies across the 20 trials only in the B6 mice. **h**, **i** The spatial strategy use in the B6 mice was significantly correlated with the dHPC volume (B6: *n* = 7, BTBR: *n* = 8) and neuronal density in the lateral cortex (B6: *n* = 6, BTBR: *n* = 7)

All search strategies used by animals (B6, *n* = 13; BTBR, *n* = 9, in total 660 trials) on visible- and hidden-platform days were grouped into either non-spatial or spatial categories (Fig. 5a), and the percentage of use of both categories at the end of the testing sessions (day 5) were calculated.

On the visible-platform day when the platform was cued, both groups used either non-spatial or spatial strategies more or less in a similar manner. Navigation in both groups was mainly based on DSe and DSw, particularly in trials 5 and 6. Also, non-spatial strategies including T and C in BTBR animals were more common than B6 mice in the first trials (Fig. 5b). The higher incidence of the T in the first trial in BTBR mice may interpret the higher escape latency in this group compared to the B6 mice on the visible platform day. Overall, the performance of both B6 and BTBR mice on the first day improved as testing proceeded, and no significant difference was found between groups in terms of spatial strategies use (*p* ≥ 0.05, Mann-Whitney *U*). In contrast, spatial navigation on the first invisible-platform day in both groups substantially relied on non-spatial search strategies (Fig. 5c). Although most animals in both groups displayed a poor use of spatial strategies across four trials of spatial training, the performance of the BTBR mice was evidently worse than that of B6 mice. For instance, the BTBR group continued to rely on RS and C in the last two trials, whereas B6 mice were able to reduce these strategies and display more DSe to the platform. However, the between-group difference statistically was not significant (*p* ≥ 0.05, Mann-Whitney *U*).

In both groups, the use of search strategies changed over 5 days of the platform-reversal protocol. On the first day when animals were required to locate the hidden platform in the new position, the prominent non-spatial strategy use in both groups remained relatively constant across four trials (*p* ≥ 0.05, Mann-Whitney *U*; Fig. 5d). While both groups were still dependent on DSe during spatial navigation, for the first time, the B6 group used the FS and P in searching for the hidden platform. However, despite some evidence of spatial strategy use in BTBR mice, they displayed minimal evidence of spatial improvement on the fifth day of investigation (Fig. 5e). The B6 mice, on the other hand, revealed a clear progression toward increasingly spatial strategies (e.g. DSe, FS, DSw; BTBR: 36% vs. B6: 87%, *U* = 61, *p* ≤ 0.021, Mann-Whitney *U*). It appears that these changes in search strategy selection were the primary cause of the improved performance in the B6 group on day 5.

#### Improvement rate

The improvement rates in both groups were calculated by the percentage differences between the spatial strategy use on the first and last days (Fig. 5f). A noticeable improvement (43%) indicated by a clear increase in the cumulative use of spatial strategies was observed in the B6 group with day, whereas the observed improvement rate in the BTBR mice over the 5 days of training was 5%. The observed differences were further supported by the significant negative correlation between latency and the frequency of spatial strategies in B6 mice (*r*_s_ = −0.457, *p* ≤ 0.043; Fig. 5g). The spatial strategy use in B6 group was also significantly correlated with the dHPC volume (*r*_s_ = 0.863, *p* ≤ 0.036; Fig. 5h) and cell density in the lateral cortex (*r*_s_ = 0.303, *p* ≤ 0.041; Fig. 5i). Therefore, not only a local cortical influence together with hippocampal involvement but also the shifts occurred in search strategy use in B6 group may be key determinants of the reduced latency during spatial navigation.

### Structural assessment

Topographical (gross) anatomy along with routine histology was used to compare the brains of animals (B6, *n* = 7; BTBR, *n* = 8). Also, ROIs for anatomical assessment were chosen based on the brain areas (i.e. cerebral cortex, HPC, septum, amygdala, and striatum) involved in the neuropathology of autism^38^.

#### Volume analysis

**Fig. 6:**
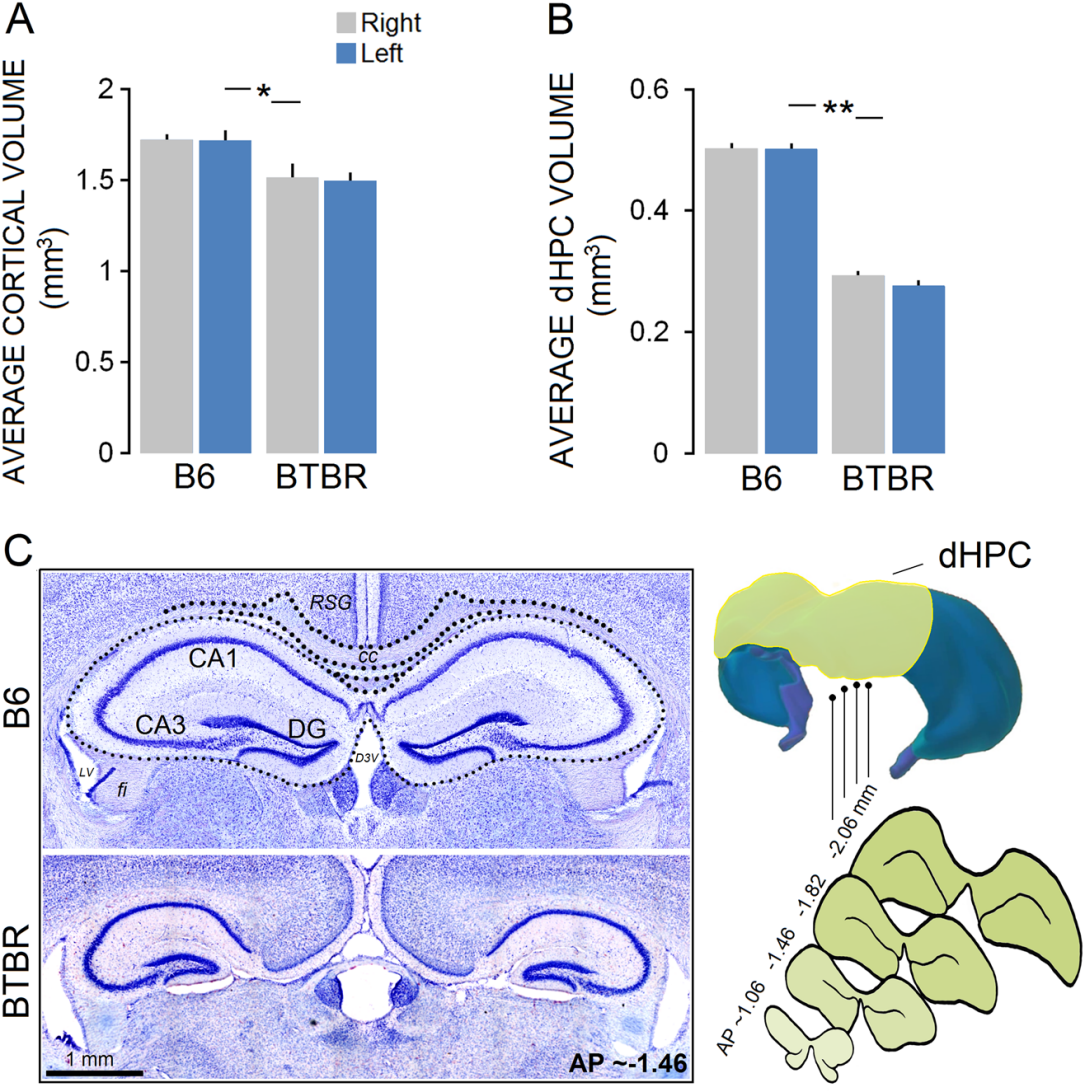
Cortical and dHPC volumes in B6 and BTBR mice. **a**, **b** BTBR mice had decreased cortical and dHPC volumes when compared with B6 mice (B6, *n* = 7; BTBR, *n* = 8). No difference was found between right and left hemispheres in any groups. **c** Left panel: Nissl-stained coronal view of the dHPC of a representative B6 and BTBR mice illustrating the area that was considered for hippocampal volumetrics. Note the callosal agenesis in the BTBR mouse and consequential lateral displacement of the dorsal third ventricle (D3V) and the lateral septum. Right panel—top: a 3D reconstruction of the dorsal hippocampus (green) in the rat brain. Right panel—down*:* a set of four cross sections of the dHPC area, from ~−1.06 to ~−2.06 mm relative to bregma were considered for volumetric analysis. Asterisks indicate significant differences: *p ≤ 0.05; **p ≤ 0.01. Error bars show ± SEM

There was no differences in brain weight, although B6 mice had slightly larger brains than BTBR animals (B6: 0.61 ± 0.07 g vs. BTBR: 0.59 ± 0.09 g; *p* ≥ 0.052). Volume analysis conducted for the left and right hemispheres showed no differences in the experimental groups (all *p* ≥ 0.05). However, the B6 mice overall had significantly greater cortical volumes (B6: 1.768 ± 0.029 mm^3^ vs. BTBR: 1.538 ± 0.028 mm^3^, *p* ≤ 0.033, Kruskal-Wallis with a mean rank cortical volume of 11.93 for B6 and 4.56 for BTBR groups; Fig. 6a) compared to BTBR mice, particularly in the prefrontal cortex (PFC; *U* = 33, *p* ≤ 0.041, Mann-Whitney *U*). Correlations between cortical volume and behavioral measures revealed a significant relationship between overall cortical volume and path speed in BTBR mice within the OFT (*r*_s_ = −0.59, *p* ≤ 0.033) suggesting that lower cortical volume in the BTBR animals was associated with greater path speed during exploration in OFT.

A comparison between groups indicated that B6 mice had larger dorsal HPC volumes by ~25% compared to the BTBR group (B6: 0.509 ± 0.012 mm^3^ vs. BTBR: 0.264 ± 0.018 mm^3^, *p* ≤ 0.001, Kruskal-Wallis with a mean rank cortical volume of 12.00 for B6 and 4.5 for BTBR groups; Fig. 6b, c). Furthermore, compared with B6 animals, most of the BTBR mice showed ventricular dilation in the lateral and dorsal third ventricles (Fig. 6c). In all BTBR animals (*n* = 8), the corpus callosum was evidently absent. Because of the clear callosal agenesis, lateral displacement in the HPC, lateral septum, and striatum was detectible in the BTBR mice (data not shown). In addition to the HPC lateral displacement, the hippocampal commissure was entirely absent in all of the BTBR mice.

#### Cortical thickness

**Fig. 7:**
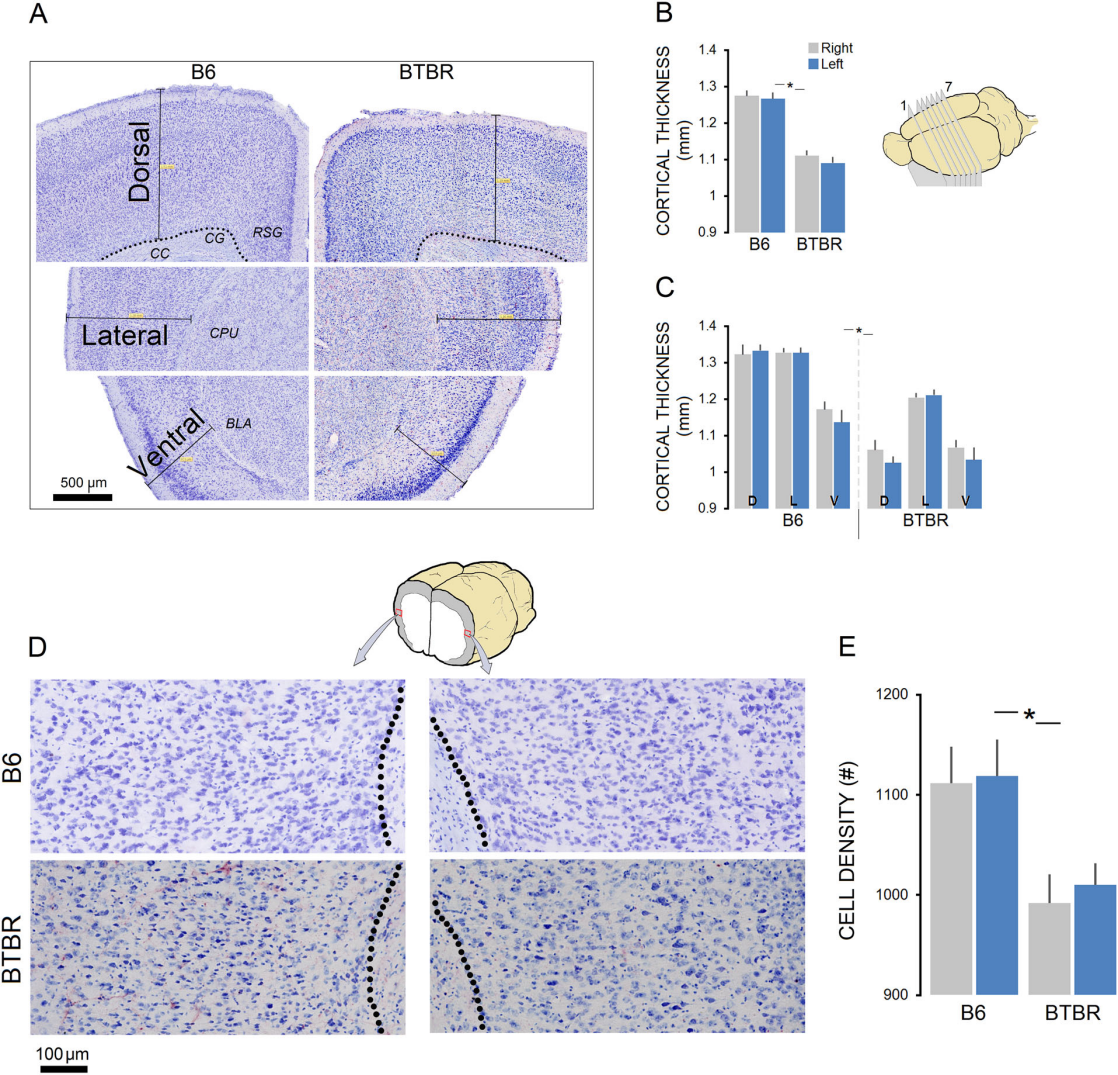
Cortical thickness and neuronal density in B6 and BTBR mice. **a** Coronal brain sections of two representative B6 and BTBR mice illustrating three cortical points (dorsal, lateral, and ventral) used for cortical thickness measurements. **b** Cortical thickness is a mean measure of both hemispheres in seven consecutive slices. B6 mice showed greater overall cortical thickness compared to BTBR group in both hemispheres. **c** The thickness of the cortex measured in the dorsal [D], lateral [L], and ventral [V] points of both hemispheres showed significant difference between groups in all regions. **d**, **e** Quantitative cytoarchitectonics-neural density shown by Nissl-stained coronal view of the lateral cortex in two representative B6 and BTBR mice. Note the differences in density of cells in both left and right regions of interests in BTBR group that is significantly decreased compared to B6 group. Red squares on left and right hemispheres represent two regions of interest in the lateral cortex that have been determined for quantitative cytoarchitectonics. Asterisks indicate significant differences: *p ≤ 0.05, Error bars show ± SEM

Cortical thickness was measured in the dorsal, lateral, and ventral points of both hemispheres (Fig. 7a). Cortical thickness was larger in B6 mice compared to BTBR group (B6: 1.270 ± 0.015 mm vs. BTBR: 1.100 ± 0.015 mm, *U* = 69, *p* ≤ 0.03, Mann-Whitney *U*; Fig. 7b). Also, there was no effect of hemisphere (all *p* ≥ 0.05), but point because ventral cortex had reduced thickness compared to other points in both groups (B6: *p* ≤ 0.021, BTBR: *p* ≤ 0.039; Kruskal-Wallis). Further analysis indicated that B6 mice had larger cortical thickness in dorsal and lateral cortices compared to BTBR animals (all *p* ≤ 0.05; Kruskal-Wallis; Fig. 7c). Also, correlation analysis between cortical thickness and behavioral variables revealed a significant relationship between cortical thickness in the dorsal cortex and stride length on the BBT in both groups (B6: *r*_s_ = 0.518, *p* ≤ 0.043; BTBR: *r*_s_ = 0.39, *p* ≤ 0.046; data not shown) indicating that dorsal cortical thickness significantly predicts stride length on the BBT.

#### Cellular density and cytoarchitectonics

The number of cells in the ROIs of the left and right hemispheres were not statistically different, and no evidence of degeneration, abnormal columnar organization, and/or gliosis was observed in any experimental groups (Fig. 7d). However, reduced cell density only in the lateral cortex of the BTBR mice (Fig. 7e) was noticeable when compared to B6 mice (B6: 1115.17 ± 36 vs. BTBR: 1001.20 ± 25; *U* = 5, *p* ≤ 0.001, Mann-Whitney *U*). No significant difference was found between groups in terms of the number of cells in the dorsal cortex (data not shown).

## Discussion

Motor deficits and cognitive impairments represent a heterogeneous array of non-diagnostic symptoms in ASD. The present experiment was directed at a detailed analysis of three lesser-known signs of ASD, sensorimotor integration (balance and coordination), locomotion, and spatial performance. These were investigated in relation to neuroanatomy in the BTBR mouse model of autism. BTBR mice displayed shorter stride length and longer latency when crossing the balance beam as compared to B6 control mice. In contrast, hyperactivity during free exploration in the OFT was apparent in BTBR mice as indicated by increased path speed and path length. Furthermore, measures of spatial memory revealed that failure to use spatial search strategies underlies the impaired performance of BTBR mice in the MWT. Neuroanatomic aberrations offered further insights into the functional deficits observed in BTBR mice; in addition to callosal agenesis and deficient dorsal hippocampal commissure (commissure of fornix), BTBR mice showed a strikingly reduced thickness and volume of cortex particularly in the PFC. Also, the HPC, measured in its dorsal part was about 25% smaller in BTBR mice when compared with their B6 counterparts.

### Impaired coordination and balance in the BTBR strain

Impaired fine motor skills in patients with ASD are strongly associated with social symptomatology^39–41^. Indeed, all three core symptoms of ASD (i.e. social interaction, communication challenges, and repetitive, stereotyped behaviors) require coordinated and synchronized neurological systems, and are established upon regulated sensory information and movement. Therefore, disturbances in motor behavior as part of autism-associated symptoms require more attention in current research.

Although ASD patients often suffer from difficulties with posture, balance, coordination, and motor planning^39,42–44^, motor impairments in the BTBR strain model of autism are minimally characterized. The lack of examination of motor deficiencies in preclinical autism models is mainly due to inconsistencies in assessment and interpretation of motor impairments in ASD compared to the general focus on communication challenges and social interaction deficits. In the present experiment, we identified a substantial motor coordination deficit and postural abnormalities (i.e. defensive-like posture with abnormally flat back, stretched trunk) that indicate that most BTBR mice moved differently than their B6 counterparts during beam walking. The reduced stride length in BTBR mice also caused a significant increase in latency on the balance beam. Apparently, reduced stride length on the beam allowed animals to maintain their center of gravity firmly within the fixed base of support. Also, longer crossing time on the beam arguably reflects a tendency to reinforce gait stability, issues that may emerge from difficulties in balance, low muscle tone (hypotonia), and even problems in proprioception. Comparable aspects of balance and coordination issues along with fragmented skilled walking patterns have been previously reported in numerous studies with clinical populations^45–49^. For instance, children with ASD exhibited abnormal limb movements, difficulties with balance, shortened steps, and increased stance times^39,47,50^. Interestingly, impairments in motor skills in ASD (e.g. deficient skilled motor gestures) result in difficulties of visual feedback integration to adjust and guide spontaneous skilled movements^45^.

Neural substrates of motor impairments in ASD include abnormalities in cerebellum and basal ganglia^51–53^. At cortical levels, however, clinical studies have shown signs of cortical thinning and neuronal loss, with no changes in overall brain volume in ASD patients^54–56^. In agreement with these findings, we report here that both dorsal and lateral cortices were thinner in BTBR mice as compared to B6 mice, while the ventral cortex remained unaffected. Also, volumetric measurements revealed no differences between BTBR and B6 groups in whole brain volume. The present correlation analysis indicated that cortical thickness in the dorsal cortex may significantly predict stride length on the balance beam. It is noteworthy that dorsal and lateral cortical areas defined for the thickness measurement in the present study mostly covered motor cortex, both somatosensory (S1) and motor cortices including primary (M1) and secondary (M2) motor subregions.

The motor cortex, in general, plays an essential role in skilled movement, postural control, and balance^57,58^. Close dialog between the M1 and S1 is essential not only for skilled motor function^35,59–61^, this intracortical cooperation is also supported by a wide range of afferent inputs emerging from extracortical and subcortical processing systems such as cerebellum^62^, basal ganglia^63^, and brainstem^64^. Although the cause of the reduced cortical thickness in the BTBR strain is unclear, such extensive morphological abnormality may be expected to severely disrupt sensorimotor integration, motor planning, and fine motor execution. Therefore, as a morphological hallmark of an undeveloped brain, cortical thinning in the BTBR strain^65^ reveals some key aspects of the neuropathology that may need further investigation in preclinical models of autism, particularly in relation to the non-diagnostic symptoms.

### Deficient locomotion and affective state in the BTBR strain

Individuals with ASD are often diagnosed with poor emotionality^66,67^. Also, many behavioral symptoms such as inattention, hyperactivity, and impulsiveness seen in children with attention deficit hyperactivity disorder are also shared with ASD^68^. In mouse models of autism, emotion-relevant activities (e.g. anxiety-like and/or avoidance behaviors) are typically assessed based on the time spent in open vs. closed arms in the elevated plus maze. Accordingly, BTBR mice make more entries into the open arms, which has been repeatedly interpreted as lower level of anxiety compared to other strains^2,69,70^. However, such interpretations can be confounded by potential artifacts due to the enhanced spontaneous behaviors such as nonspecific hyperactivity and impulsiveness. The OFT in the present study was employed to rule out these confounding effects.

During free exploration in the OFT, BTBR mice moved substantially faster than B6 controls. This finding that stands in contrast with previous reports^5,8,71^ possibly due to differences in test procedures (e.g. size of open field) and the age of animals. Also, the BTBR strain showed no reduction in exploration speed even in the last 5-min time bin interval, whereas the B6 mice exhibited significantly lower speed scores in the same period as compared with the first time bin interval. This indicates that BTBR mice failed to habituate to the arena. Hyperactivity, even in the central zone of the OFT, also can show impaired response inhibition (RI)^72^ in the BTBR strain, as previously seen in clinical autism^73^ (see also ref. ^74^ for review). Therefore, the failure to habituate to the physical characteristics of the OFT along with inadequate inhibition of movement and arousal portrays a cardinal feature of ASD by which high levels of arousal lead to labile attention in the BTBR animals.

A puzzling feature of the present findings concerns the differences in overground locomotion by which BTBR mice moved faster than their B6 counterparts in the OFT while they were slow and relatively accurate on the BBT. As proposed for individuals with ASD^73^, the impaired RI in the OFT and intact RI on the BBT for BTBR mice support the notion that inhibitory processes seen in clinical ASD cases may be task-dependent. A second implication of these findings is that non-diagnostic symptoms (e.g. impaired movement, poor coordination, hypo- or hyperactivity, and subtle deficits in spatial cognition associated with learning difficulties), which belong to domains outside the major diagnostic triad of ASD may be impacted in similar patterns than traditional diagnostic signs.

The reduced cortical volume may contribute to the behavioral abnormalities observed in this study. Although measures of cortical volume alone may not be sufficient to decisively explain the existing impaired RI and hyperactivity in the BTBR strain, cortical volume reduction in the present study could robustly predict higher speed of the BTBR mice in the OFT compared to B6 animals. The reduced cortical volume and thickness found in BTBR mice also may be indicative of decreased functional capacity of cortico-cortico (e.g. frontal and posterior cortical circuits) or cortico-subcortical (e.g. fronto-striatal pathways) connections. Though it was not the focus of this experiment, hyperactivity along with repetitive behaviors in ASD can likely be attributed to disordered cortical connectivity^75^ that may produce excessive excitability, or to an impaired coherence across the cortico-striatal circuits in several interconnected brain regions^76^. This connectivity repertoire, therefore, appears to play an elemental role in both bottom-up and top-down modulation of executive control, including that involved in modulation of visuo-motor control and RI in a particular task.

### Spatial memory challenges in the BTBR strain

It is difficult to describe a uniform picture of spatial prowess and navigational processing in ASD on the basis of the extant literature. However, there is a growing number of claims that spatial cognition (e.g. goal-directed spatial performance), in parallel with social behaviors and locomotion, is impacted by ASD^77^. Previously, the MWT was used to examine some aspects of perseverative behaviors or insistence on sameness in the BTBR strain^2^. However, the BTBR strain’s cognitive phenotype, especially spatial learning and memory is not well characterized. In the present study, we used the MWT to measure spatial performance in BTBR mice with further focus on the analysis of search strategies. We observed pronounced differences between BTBR and B6 strains in either traditional measures of spatial performance or search strategies used by animals to locate the hidden platform in the MWT. Our observations showed that goal-directed spatial behavior, which is critically HPC-dependent in humans and rodents^78–80^ was extremely impaired in the BTBR mice. All traditional indicators of spatial behaviors in the task support this conclusion. Furthermore, the BTBR strain did swim faster in both visible and hidden-platform versions of the task as compared to B6 animals. The fast searching behavior, however, could assist in decreasing the latency only in the visible-platform protocol as well as initial stages of spatial testing. More importantly, a combination of these conventional readouts and search strategies for the first time in the BTBR strain provided further explanations on many other aspects of impaired spatial function of these mice that would otherwise have not been easy to interpret. This approach was chosen to differentiate between HPC-dependent allocentric and HPC-independent egocentric search strategies.

The analysis of search strategies has previously been successful in elucidating the dynamics of spatial performance in transgenic mice^31^ because it was shown to be dependent upon the HPC structural and functional integrity^26,30^. The present study determined that BTBR mice used less effective strategies compared to B6 mice, a cognitive inflexibility that can account for the correlation between dHPC volume and the frequency of spatial search pattern. BTBR animals also displayed minimal evidence of an actual spatial reference memory in the probe trial. Furthermore, when animals were required to locate the hidden platform in a new quadrant (reversal learning protocol), search strategy use in the BTBR mice did not significantly improve toward spatial patterns with 5 days of intensive training. Interestingly, there was less evidence than expected for repetitive or stereotyped behaviors in BTBR mice that might have been shown by unusual T, and/or circling and C strategies during navigation in the MWT. Overall, while B6 mice reduced the use of non-spatial strategies across spatial testing, and their navigation to the hidden platform remarkably relied upon spatial strategies (~87%) on the last day of the 5-day reversal learning period, the BTBR strain failed to exhibit the same profile of improvement (~36%).

The neuroanatomical basis for differences in search strategy is not well known. Basically, spatial processing and behaviors are determined by the integration of information and coordination of multiple neural systems such as PFC and HPC. Moreover, spatial performance is proportional to the volume of HPC spared^81^. In the domain of structural defects in clinical autism, smaller HPC volume has been reported in several studies^82,83^. Nevertheless, the HPC abnormalities, either structural or functional are somewhat heterogeneous. Some studies suggest that there are differences in HPC volume and shape^84^, as well as HPC connectivity^85^ between patients with ASD and controls, whereas other studies have not found the same differences^86^.

Anatomical abnormalities related to spatial challenges may not be only limited to HPC dysfunction. A nearly complete absence of corpus callosum and hippocampal commissure in the present BTBR cohort may deprive the central processing system from an inter-hemispheric synchronized dialog^87^ required for noncortical synchrony and integrated HPC-dependent behaviors in a given task. Regarding the callosal and commissural abnormalities, reduced dHPC volume, and the poor use of spatial search strategies in BTBR mice, it would not be unreasonable to assume that a global disorganization in HPC anatomy, arguably together with decreased cortical cell density, volume, and thickness may be responsible for disrupted search strategy use.

## Concluding remarks

It is widely acknowledged that ASD uniquely appears in humans. Nevertheless, numerous research attempts have been made to mimic the behavioral, typically diagnostic symptoms of ASD in animal models such as the BTBR inbred mouse strain. Here we sought for non-diagnostic symptoms of autism within three motor and cognitive domains in the BTBR mice, besides the traditional behavioral phenotyping. We did incorporate face validity and construct validity in our detailed analysis, and concluded that non-diagnostic autistic-like symptoms in the BTBR mouse strain can be similarly impacted by autism risk factors as the traditional diagnostic signs. We also conclude that sensorimotor disintegration, locomotor abnormalities, and impaired spatial behavior along with the corresponding neuropathology represent effective measures of severity of impairments in one or more non-diagnostic domains. These in fact may confound final conclusions about core diagnostic symptoms linked to ASD. The underlying neuropathological mechanisms for these specific, lesser-known behaviors, and how they influence diagnostic reliability in ASD still await additional investigation beyond face validity.

## Disclaimer

The sponsor had no role in the planning or conducts of the study or in the interpretation of the results.

## Electronic supplementary material

Sup Legends

Sup Mov 1

Sup Mov 2

Sup Mov 3

Sup Mov 4
