# Supplementary material for: Non-diagnostic symptoms in a mouse model of autism in relation to neuroanatomy: the BTBR strain reinvestigated
Source: Transl Psychiatry. 2018 Oct 26;8:234. doi: 10.1038/s41398-018-0280-x (PMC6203744; doi:10.1038/s41398-018-0280-x)
Supplement: Supplementary file 1 — Sup Legends [file 41398_2018_280_MOESM1_ESM.docx]

**Supplementary Legends:**

**Supplementary Movie 1**: Movie of the last trail of a B6 mouse on the Balance Beam Task (BBT). A larger stride length in the B6 group was associated with lower latency during beam walking when compared with BTBR mice.

**Supplementary Movie** **2**: Movie of the last trial of a BTBR mouse on the Balance Beam Task (BBT). Note that beam walking in the BTBR animals was characterized by shorter stride length and higher latency than B6 mice.

**Supplementary Movie** **3**: Movie of the path taken by a B6 mouse in the last 60 seconds of the exploration in the Open Field Task (OFT).

**Supplementary Movie** **4**: Movie of the path taken by a BTBR mouse in the last 60 seconds of the exploration in the Open Field Task (OFT). BTBR animals explored the arena faster than B6 group. Also, analysis of distance traveled in the OFT indicated that the BTBR group traveled significantly more distance than B6 mice.
